# Outcomes of Flapless Er:YAG and Er,Cr:YSGG Laser-Assisted Crown Lengthening: A Systematic Review

**DOI:** 10.3390/dj12120418

**Published:** 2024-12-20

**Authors:** Haitham Elafifi Ebeid, Walid Altayeb, Isabel Parada Avendaño, Daniel Abad-Sanchez, Josep Arnabat-Domínguez

**Affiliations:** 1 Faculty of Medicine and Health Sciences, University of Barcelona, 08907 Barcelona, Spain; dabad@ub.edu; 2 Surgery Department, Master of Oral Laser Applications (EMDOLA), University of Barcelona, 08908 Barcelona, Spain; 3 Department of Periodontics, Faculty of Dental Medicine, Istanbul Aydin University, Istanbul 34390, Turkey; draltayeb@hotmail.com; 4 Bioestatistics Department, Analysis and Design in Clinical Investigation, University of Barcelona, 08908 Barcelona, Spain; cotihums@gmail.com; 5 Idibell Institute, 08908 Barcelona, Spain

**Keywords:** Er:YAG laser, Er,Cr:YSGG laser, flapless crown lengthening

## Abstract

Introduction: In recent years, erbium-doped yttrium aluminum garnet (Er:YAG) and erbium, chromium/yttrium–scandium–gallium–garnet (Er,Cr:YSGG) lasers have been introduced as another possibility to perform less-invasive flapless (FL) crown-lengthening (CL) procedures. Objectives: The aim of this review is to describe the outcomes and complications of this approach. Materials and methods: A literature review was conducted to retrieve clinical studies and case reports that analyze different variables related to laser-assisted flapless crown lengthening and report their outcomes in terms of gingival margin level stability (GMLS), and postoperative complications. Results: A total of five studies were included in the final qualitative analysis; two of them were randomized controlled trials (RCTs) and the rest were case reports. The common variable measured in all studies was the GMLS, finding good stability in the FL groups at 3 months follow-up, but more tissue rebound was observed in patients with the thick biotype. Other variables were reported in different articles as the plaque index (PI), gingival index (GI), bone margin level, biotype, bleeding on probing (BP), probing depth (PD), and postoperative pain by the numeric rating scale (NRS). Discussion: There are a wide range of heterogenous clinical variables used to evaluate outcomes, as well as variations in the type of laser used and its parameters in terms of the applied technique. However, most analyzed studies showed better GMLS for the flapless technique, as well as less postoperative inflammation. Conclusions: The included studies showed promising clinical outcomes in the FL laser-assisted CL groups concerning GMLS at the 3-month postoperative period. However, more RCTs are needed with respect to fixed laser parameters and patient biotype selection to reach a definitive clinical protocol.

## 1. Introduction

Surgical crown lengthening (CL) is a technique used to gain clinical crown height. It may be needed for esthetic purposes due to the presence of excessive gingival tissue, or to achieve a more harmonious and symmetrical gingival contour such as in cases of altered passive eruption, hereditary gingival fibromatosis, drug-induced gingival enlargement including from anticonvulsants, immunosuppressants, and calcium channel blockers, and reactionary gingival enlargement when the altered passive eruption is aggravated by mouth breathing [1]. Another indication is for functional purposes, to restore short clinical crowns or a badly decayed tooth. The conventional technique involves a gingivectomy, which consists of soft-tissue gingival margin recontouring to the ideal or desired level, followed by osseous resection to reshape the bone, creating an adequate biological width after flap elevation, and finally, apical repositioning of the flap and suturing. Numerous instruments have been proposed for performing the osteotomy stage in CL. These include the use of osteotomes and mallets, rotatory devices, micro-saws, and piezoelectric tools [2,3]. However, this technique can be associated with postoperative inflammation, bleeding due to the heat generation and mechanical trauma, and a sometimes unstable final gingival marginal level [4].

Minimally invasive flapless (FL) crown lengthening has been proposed for a specific indication without requiring the reflection of a flap, using a scalpel for the gingivectomy and micro-chisels for the osteotomy through the incision.

This technique eliminates the need for flap repositioning and suturing, which can alter the final gingival marginal level stability (GMLS) due to flap mobilization [5].

In a systematic review and meta-analysis, Crosby et al. [6] showed the superiority of the FL piezo surgery in terms of reducing surgical trauma, chair time, postoperative pain, and accelerated healing, while maintaining a stable gingival margin at 12-months follow-up. 

**Figure 1:**
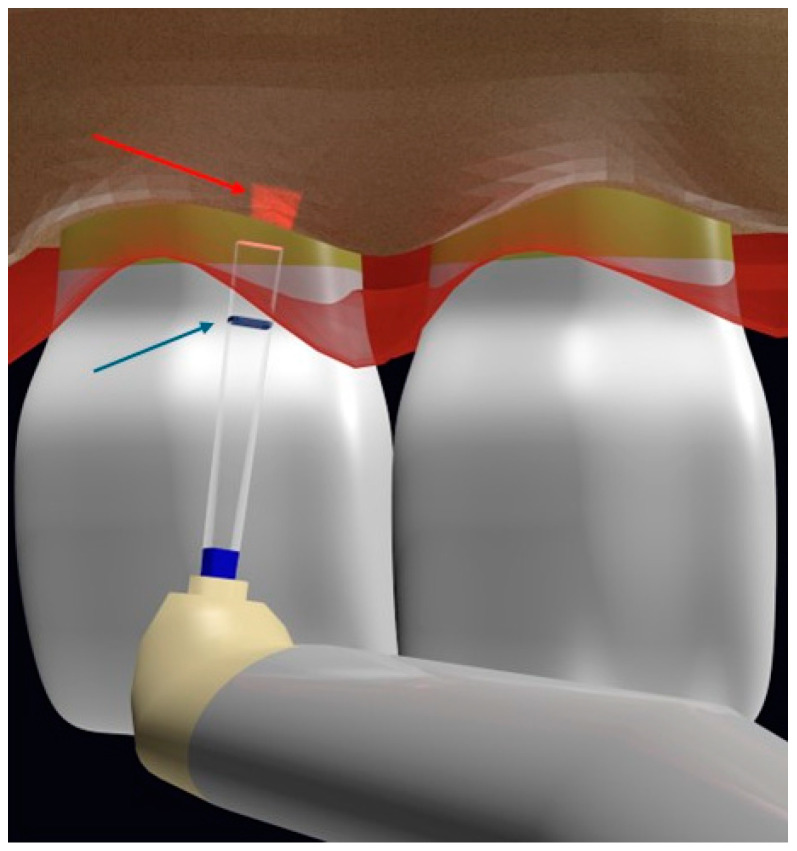
Illustration of the Er,Cr:YSGG osteotomy with chisel tip using the flapless technique marking the depth in the laser tip (blue arrow), showing the aiming beam on the alveolar crest (red arrow) (authored by Dr Elafifi).

Based on this idea, several authors [7,8,9] reported the possibility of performing a minimally invasive FL CL using all-tissue lasers, with the advantage of bone contouring through the gingival sulcus marking the desired cutting depth on the laser tip and also without the need to reflect a flap (Figure 1).

Er:YAG (2940 nm) and Er,Cr:YSGG (2780 nm) lasers have the capability of cutting both hard and soft tissues with exceptional surgical precision and minimal collateral thermal damage, making them ideal for performing minimally invasive CL. This approach reduces tissue damage and minimizes bleeding, inflammation, and postoperative discomfort, thereby promoting improved healing outcomes [10,11,12].

**Figure 2:**
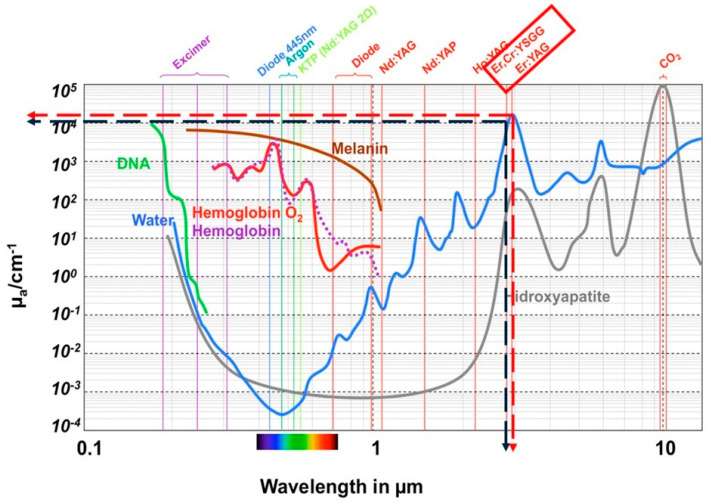
The coefficient of absorption of different wavelengths by various tissues (the red and black dotted arrows show the wavelengths of Er:YAG (red dotted line) and Er,Cr:YSGG (black dotted line) represented on the y-axis and their water absorption coefficients are measured in µ_a_/cm^−1^ (x-axis).

The erbium family of lasers (Er,Cr:YSGG and Er:YAG) has shown a high absorption coefficient for water molecules compared to other laser wavelengths [Figure 2], which makes them suitable for both soft- and hard-tissue ablation with minimal thermal damage.

In contrast to rotary instruments that generate more friction, vibration, and thermal damage, laser-assisted procedures may reduce the amount of local anesthetic used [13] and there is a reduced need for water spray, which enhances visibility and makes the surgical procedure easier.

One of the main advantages of the use of erbium-doped lasers in bone cutting is less smear layer generation, which results in accelerated healing and less postoperative discomfort. In an animal study, Beak et al. [14] compared the clinical effect of bone cutting using piezo surgery in one group and the Er:YAG laser in other group based on a scanning electron microscopic analysis, demonstrating fresh bleeding patterns in laser osteotomies, which indicates a cleaner cut with less smear layer generation; this was confirmed by the scanning electron microscopy (SEM) observation that showed more surface cracking and smear layers in the piezo group compared to the more open and intact bone surface of the laser group.

In a previous study, Er,Cr:YSGG lasers showed effective bone ablation with minimal thermal damage and no evidence of surface melting. An unaltered calcium/phosphorous ratio was also observed, which indicates no chemical alteration of the surrounding bony tissues after ablation [15].

The erbium family of lasers has demonstrated effectiveness in several periodontal procedures with minimal invasiveness. In one study, the use of an Er,Cr:YSGG laser in non-surgical periodontal treatment seems to achieve the same results when compared to the mini-flap procedure. But, the laser group showed better patient perception [16].

To date, there is still limited evidence regarding the long-term outcomes when using the FL laser-assisted CL technique as a routine clinical procedure. There are several factors to consider when evaluating this novel technique, including the technical difficulty of the procedure, gingival margin stability, and overall quality of the newly attached periodontal apparatus.

The aim of this systematic review is to describe what evidence the literature provides us regarding laser-assisted FL CL, using either Er:YAG or Er,Cr:YSGG lasers, in terms of its outcomes in achieving GMLS as well as overall periodontal stability, the possible procedural errors of this technique, and postoperative pain and inflammation compared to the conventional technique.

## 2. Materials and Methods

We carried out this systematic review following the PRISMA guidelines. The main question of our research was as follows: Can closed-flap Er:YAG or Er,Cr:YSGG laser-assisted crown lengthening be considered a predictable and reliable technique with similar or superior outcomes compared to the conventional technique in terms of overall periodontal tissue stability?

The PICO tool was used to formulate our focused question as follows:

Population (P): Adult patients ≥ 21 years old (after the completion of passive eruption) who require functional or esthetic crown lengthening that involves both soft- and hard-tissue remodeling in any teeth group.

Intervention (I): Flapless Er:YAG or Er,Cr:YSGG laser crown lengthening (soft- and hard-tissue ablations).

Comparison (C): Open flap (OF) crown lengthening using conventional rotary instruments and an all-tissue laser (Er:YAG or Er,Cr:YSGG).

Outcomes (O): GMLS and tissue rebound, and complications if reported.

Search strategy:

H.E and I.P independently carried out an electronic search, and harmonization was carried out by J.A. in case of a non-consensus between the 2 authors. The inclusion criteria were articles written in the English language, human RCTs, and case series or case reports involving FL CL with a minimum follow-up of 3 months. All studies that reported only laser gingivectomy without osseous resection were excluded.

**Figure 3:**
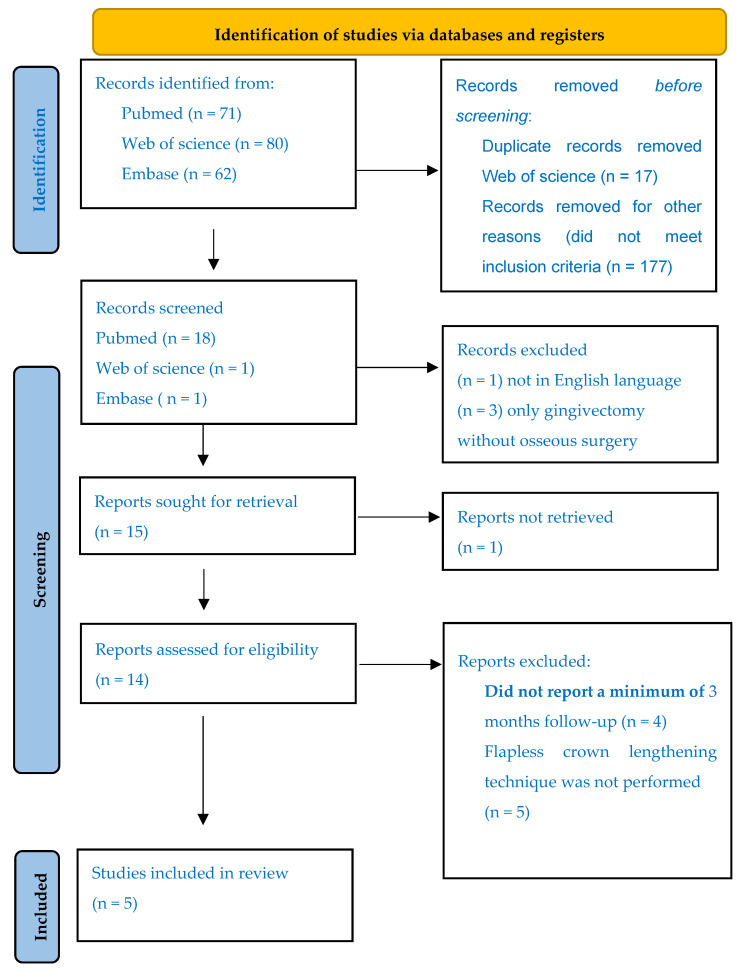
PRISMA flowchart.

The search was carried out with no date restriction for studies published up until the year 2024 in PubMed with an advanced search using the MeSH terms ((crown lengthening) AND (lasers)) (71 results); studies were screened to select human studies (RCTs and cases series or case reports) that used erbium-doped lasers to perform closed-flap crown lengthening. We also searched the Web of Science (WOS) database, finding a total of 80 articles in this database. Embase was also searched, finding a total of 62 studies. Duplicated articles were removed (17 studies) and 177 did not meet the inclusion criteria. After screening 19 studies, we eliminated 3 articles because they only involved a laser-assisted soft-tissue procedure, and 1 study was not in English language. A total of 15 reports were sought for retrieval and 14 papers were retrieved and assessed for eligibility. A total of 5 articles were excluded due to the lack of the flapless CL procedure and 4 studies did not report the minimum 3-month follow-up. Five articles were included for the final qualitative analysis (Figure 3).

Data collection:

The main outcomes evaluated were GMLS, supracrestal gingival tissue dimension, and postoperative pain and inflammation if reported.

## 3. Results

A total of five studies were included in the final qualitative analysis. Regarding the study type, only two randomized controlled clinical trials were found [7,9], and the remainder included one case report [17] and two case series [18,19].

**Figure 4:**
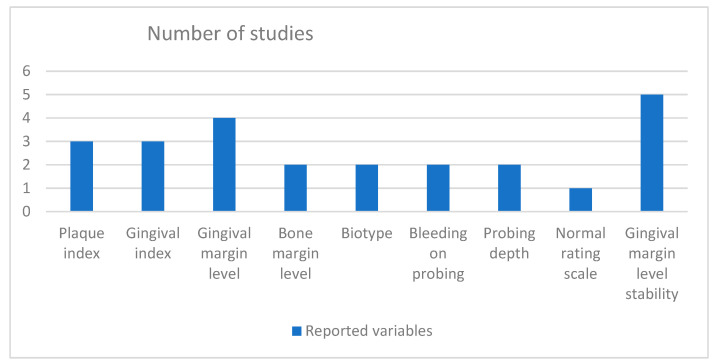
The different variables measured among the included studies.

The variables measured among the studies were plaque index (PI), gingival index (GI), gingival margin level, bone margin level, biotype, bleeding on probing, and probing depth. Only one study examined pain level using the numeric rating scale (NRS) on the first and seventh postoperative day [9]. The GML stability was the commonly reported variable in all of the included articles (Figure 4). 

Regarding the type of the laser used, two studies used an Er,Cr:YSGG laser [7,9] and the remaining three studies used an Er:YAG laser [14,15,17].

The following variables were analyzed in the included studies:

GMLS and tissue rebound:

Two authors [7,9] reported better GMLS at 3-months follow-up in the flapless laser group. Altayeb et al. [7] reported more tissue rebound in the open flap (OF) group at 3-months follow-up with no statistically significant difference at 6 months compared with the control; they also stated that tissue rebound is likely in the thicker phenotype. Nevertheless, Chen et al. [18] did not report tissue rebound in their case series. The rest of the authors did not specify GMLS in their results.

Follow-up periods:

Although all the authors of the included studies reported a minimum follow-up period of 3 months, the mean minimum follow-up time among the articles was 2.60 months (SD = 2.29), while the maximum was 13.2 months (SD = 13.17).

Laser parameters for bone ablation:

The authors who applied the Er,Cr:YSGG laser used between 3.5 [9] and 4W [7]; only the latter mentioned the frequency (20 Hz) and the pulse duration (60 µs). The studies that involved the Er:YAG laser reported the energy per pulse in the range of 50–70 mJ [17,18,19]. All of them reported the use of water spray irrigation.

Data extraction from the included studies was performed (Table 1) for the following variables: author and year of publication, study design, randomization (if applicable), number of patients, age group or mean age, type of laser used, laser parameters for both soft and hard tissues, treatment groups (if applicable), periodontal variables studied, follow-up periods, and results reported by the authors. We also classified those studies regarding the grade of recommendation and level of evidence (Table 2).

**Figure 5:**
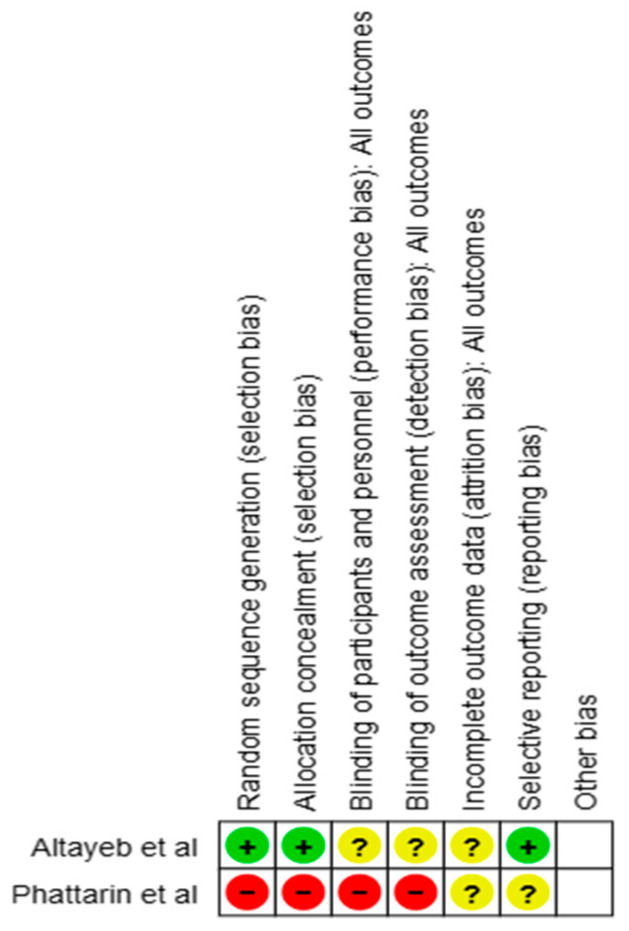
Risk of bias assessment (green color indicates low risk of bias, yellow color indicates unclear risk of bias and red color indicates high risk of bias) [7,9].

Risk of bias analysis was performed on the two controlled clinical trials included using the Cochrane ROB tool 2.0 (Figure 5).

## 4. Discussion

The effectiveness of minimally invasive dental treatments is under continuous investigation, which aims to reduce the morbidity of the procedures used and improve patients’ quality of life. Laser technology has shown very promising results in its use in several soft-tissue procedures such as gingivectomies, frenectomies, and oral mucosa biopsies, with minimal intra- and postoperative discomfort [20,21].

Surgical CL can be an essential step in functional or esthetic rehabilitation. GML stability is crucial to achieve a successful outcome after CL, especially when prosthetic rehabilitation is considered [22].

Gingival tissue rebound is a challenging problem after a conventional CL procedure, as reported by Pilalas et al. [23] in a systematic review concluding shortening of the gained clinical crown after a 3-month period postoperatively as a common finding. This can be related to several factors such as the patients biotype (more rebound in the thick phenotype), tooth position, amount of osseous recontouring, and flap elevation beyond the mucogingival line, which increases the flap mobility leading to an unstable final position after flap suturing [24,25,26]. Attempts to precisely control the exact amount of soft tissue and the bone needed to be removed using a surgical guide were proposed, but there was no significant difference in terms of postoperative tissue rebound because a flap was still raised as in the conventional way [27].

The conventional method involves flap elevation, which makes the predictability of the final position of the GML difficult to determine on repositioning the flap. Moreover, the amount of bone removal in length and thickness is difficult to assess to obtain soft-tissue stability. The Chu aesthetic probe has been suggested to determine the ideal crown proportion and amount of bone resection needed to preserve the biological width [28], but this technique has limitations because it uses standardized measurements for all the patients. Biological width determination for each patient is important to achieve a stable postoperative GML [29]. Hamasni et al. [30] reported the variability of this measurement depending on tooth location, probing site, and gender in a clinical study. Domínguez et al. [31] showed a more stable GML up to 6 months when the flap elevation does not extend apical to the mucogingival line and the gingival margin lies ≥ 3 mm to the osseous crest. However, some limitations were found in this study such as the lack of a control group, lack of biotype determination before the intervention, and the surgical procedures being carried out by different operators. More tissue rebound interproximally can be a common finding in an OF procedure due to the maintained buccolingual bone width in these areas [32], since a direct correlation between bone thickness and coronal gingival tissue creeping has been found [5]; there is still a lack of predictability for the final GML in the OF cases.

Another important factor is the supracrestal tissue dimension that can be calculated clinically before performing the CL by subtracting the sulcular depth from the bone sounding. Hamasni et al. [30] achieved maximum tissue stability in a clinical study when the supracrestal tissue dimension was maintained after surgery compared to the preoperative measurements. This factor is essential to determine the amount of bone removal needed. Nevertheless, in most cases, when esthetic rehabilitation is planned, the amount of both soft- and hard-tissue removal depends on the smile design and facial analysis, which can neglect the individual biological considerations. 

In recent years, an FL approach has been introduced that differs from the conventional technique in its lack of flap elevation and suturing, which makes the level of the gingivectomy more precise and controls for future prosthetic rehabilitation with less patient morbidity. However, one of the limitations of this technique is its contraindication in cases of inadequate, keratinized, attached gingiva where an apically repositioned flap or a previous mucogingival surgery might be indicated.

A wide range of heterogenous parameters have been examined in existing studies, but we focused on outcomes, possible complications, and postoperative pain and inflammation. The FL technique is thought to achieve more GMLS. Phattarin et al. [9], in a controlled clinical trial, reported greater gingival margin stability at the 3-month follow-up in the Er,Cr:YSGG laser closed-flap group compared to the conventional surgery group. This result is in accordance with that reported by Altayeb W. et al. [7] in an RCT using the same type of laser, comparing FL with OF procedures at 1-, 3-, and 9-months follow-up. They found better healing and GML stability in the FL group only at 3 months, with no differences between groups at 9 months. The recommended minimal follow-up period to judge GML stability is usually 3 months, as seen in previous studies [7,9,18]; this is because, at this time, elevated levels of osteoprotegerin can be found in cases of open flap procedures, which is associated with an intensified bone remodeling process [5].

Biotype determination of patients’ gingival tissues is very important for case selection and treatment planning. It can be determined, according to Forst et al., by the periodontal probe visibility through the gingival tissue on probing [33]. Other methods such as that shown by Altayeb et al. [7] used cone beam computed tomography (CBCT) to measure the gingival thickness from the crestal bone up to 2 mm apically to determine the biotype in a quantitative manner. Also, they utilized the CBCT to control the alveolar bone height and contour, as well as the gingival supracrestal tissue dimensions at 9 months. Two studies reported more tissue rebound in the thick gingival biotype [7,21]. Nevertheless, Chen et al. [18] did not detect changes in the GML at 3 months after Er:YAG laser-assisted CL in their case series. This contradictory result can be related to the method of tissue stability evaluation, since these authors did not use a template as a reference and depended only on probing.

Tissue rebound can also depend on the tooth type and site, presence of restorations, and overall periodontal health. Carneiro et al. [34] showed the difference in tissue rebound depending on the tooth type in a 12-month follow-up after an OFL conventional technique; they reported more tissue rebound in the central incisors (mean average of 0.75 mm), followed by the canines (0.65 mm), while the lateral incisors showed the most GMLS. In our review, there was heterogenicity between the teeth groups in the different studies; four authors [7,17,18,19] reported CL on the maxillary anterior teeth, three of them performed prosthetic rehabilitation afterwards [7,18,19], one author [17] performed only the CL, and one report [9] was based on functional CL on posterior teeth for restorative purposes without specifying the exact teeth groups, reporting no tissue rebound at 3 months. It seems that more GML stability can be achieved with the FL technique even when individual biological width is not assessed in each case and performed by 2.5–3 mm based on the average values.

Addressing the complications that can occur, Michael K et al. [19] was the first author to reflect a flap after performing the FL laser-assisted technique in a case series to study the possible complications associated with it. They reported the presence of osseous troughs as a common finding, which required further recontouring to establish a healthier attachment apparatus, especially in the esthetic zone, to achieve a positive bone architecture. This problem was addressed in the study of Altayeb W. et al. [7] by changing the orientation of the chisel laser tip parallel to the osseous crest and inclined away from the root surface to eliminate them, this is the same tip used by Michael et al. [18] to remove bony troughs after flap reflection. However, the areas with thicker buccal bone can be more challenging for the FL technique due to the lack of direct visibility and difficulty in determination of the quantity of bone removal in terms of thickness, since leaving a very thin buccal plate can lead to dehiscence, fenestrations, and more crestal bone resorption, which can alter the final position of the GML [35].

**Figure 6:**
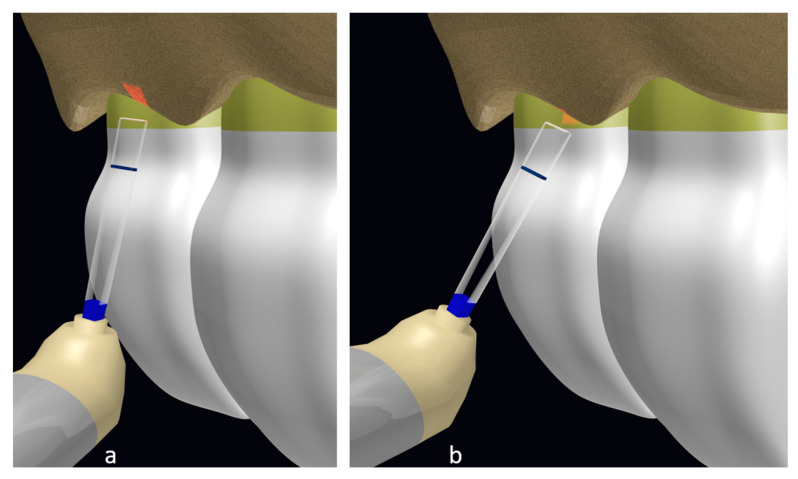
(**a**) Correct angulation for bone ablation and (**b**) incorrect orientation that can cause root surface pitting (authored by Dr. Elafifi).

Moreover, Michael K et al. [19] reported root surface pitting in two cases due to the misdirection of the laser tip toward the root surface, as demonstrated in the illustration (Figure 6); this finding was also reported by Chen CK et al. [18]. In relation to this, Altayeb W. et al. [7] modified their technique to provide a safer and more efficient osteotomy by directing a prism chisel tip in a more parallel manner to the tooth surface, together with controlled laser parameters by using short pulse duration (60 µs) and lower pulse frequency (20 Hz) with more water irrigation. The CBCT they performed at 9 months post-operation did not detect any signs of iatrogenic problems related to the surgical technique.

Regarding the postoperative pain outcome, not all authors reported this variable. Phattarin et al. [9] measured pain levels using NRS at 1 and 7 days post-operation, showing lower pain levels in the laser group compared to the conventional surgery. Nevertheless, Chang-kai et al. [18] indicated that the administration of non-steroidal anti-inflammatory drugs as necessary, but they did not report the quantity consumed by the patients in each group. The lower pain levels can be attributed to the minimal trauma induced by the laser and more precise ablation. The minimal trauma theory is supported by histological analysis of excised gingival tissues, indicating a necrosis zone of only 35–70 µm, which corresponds to 5–10 tissues deep [18]. Rebeiro et al. [5] compared OF and FL procedures using conventional instruments in both groups and found no difference in terms of postoperative pain or discomfort at 7-day and 6-month postoperative follow-ups; this confirms the minimal trauma theory of lasers and their ability to reduce the morbidity of the procedures.

A metanalysis could not be conducted due to the limited number of homogenous randomized controlled clinical trials on this topic. To date, there is still a lack of a clear protocol and parameters for this approach due to the variations in different types of lasers. For example, Er:YAG lasers have a 300-times-higher water absorption coefficient than Er,Cr:YSGG lasers, which can lead to variations in the parameters to be used. Lower energy densities are needed in the case of Er:YAG lasers to achieve the same amount and rate of hard-tissue ablation. The Er:YAG laser generates less thermal effect than the Er,Cr:YSGG laser, as found in the ex-vivo study by de Oliveira et al. [36]. However, the thermal effect was limited to the superficial area compared to the bur cut, which generates heat in deeper tissues. In the reviewed studies, only one author reported the postoperative NRS for pain levels, so we cannot correlate this variable between the studies with different wavelengths.

Regarding the energy density or fluence, it is the amount of laser incident energy per area of spot size and is a very important parameter to determine the rate of tissue ablation per pulse since it determines the degree of impact of the laser energy with the target tissue to produce the photo-disruptive effect. It is also essential for study reproducibility; nevertheless, this parameter was reported by one author (148 J/Cm^2^ for ostectomy) [7]. Another study [19] mentioned the energy per pulse and the tip diameter and we were able to make an approximate calculation of the fluence (26.5 J/Cm^2^) using the area of the tip they used. This calculation is not accurate because the tip is not used in contact but at a 2–3 mm distance, which can vary the spot size. Also, two authors [17,18] mentioned only the energy per pulse without reporting the tip diameter and, finally, one study [9] did not mention either the energy per pulse or the repetition rate, so it is not possible to deduce the fluence. In general we can notice that higher energy densities are necessary to achieve bone ablation in the case of the Er,Cr:YSGG laser compared to the Er:YAG laser due to the different water absorption coefficients between them. However, the clinical impact on this difference in terms of postoperative pain or inflammation is still lacking in the literature.

With regard to the limitations, the main one is the lack of a complete laser parameter description in the articles such as the laser tip geometry, diameter, and material used; the average power settings; the pulse duration; the pulse frequency; the laser energy per pulse; and the energy density, water, and air percentage. However, the biotype and tooth type treated (incisors, premolars, or molars) can bring a variability in the outcomes just like biological width determination as another factor that could vary between patients instead of taking the 3 mm range as a rule to guide the procedure.

In the end, a good report and standard evaluation method allows facilitation of the reproducibility of the methodology, comparing results and offering a reliable clinical protocol.

## 5. Conclusions

Based on the few clinical studies and case reports, lasers have several advantages such as reduced intraoperative trauma and bleeding, a shorter operative time, and a more favorable postoperative recovery in terms of pain levels.

Some problems resulting from the lack of sufficient visualization, such as root surface pitting or leaving bony troughs, could present challenges for achieving an optimum outcome and GML stability.

## Figures and Tables

**Table 1:** Data extraction from the included studies (W: watt, Hz: Hertz, µs: microseconds, W%: water percentage, A%: air percentage, J: joules, mJ: millijoules, Cm: centimeter, PD: probing depth, PI: plaque index, BOP: bleeding on probing, GI: gingival index).

Author	Study Design	Randomization	Number of Patients	Age Group	Laser Used	Soft-Tissue Parameters	Hard-Tissue Parameters	Treatment Groups (If Available)	Variables Studied	Follow-Up	Results
Walid Altayeb et al., 2022 [7]	RCT	Adaptive randomization by changing the allocation probability according to the progress and position of the study to minimize intergroup imbalance	36	22–45 years old	Er,Cr:YSGG	To mark the cut: MT4 tip, 1 W, 50 Hz, 700 µs pulse, 43 J/cm^2^, 10% W, 10% A Gingivectomy: MZ6 tip, 3 W, 50 Hz, 700 µs, 57 J/cm^2^, 40% W/20% A. Incision flap: MT4 tip, 2.5 W, 50 Hz, 600 µs, 106 J/cm^2^, 20% A, 20% W.	Osteotomy MC3 tip, 4 W, 20 Hz, 60 µs, 148 J/cm^2^, 80% W, 20% A. To smoothen the crest: MZ6 tip, 3 W, 30 Hz, 60 µs, 95 J/cm^2^, 80% W, 20% A	Flapless (FL) AND Open Flap (OF)	Gingival margin level, supracrestal gingival tissue dimension, periodontal phenotype, PI, GI, BOP, PD, CBCT.	Immediately post-operation, and 1, 3, and 9 months	GML stability significantly better in FL only until 3-month follow-up. More tissue rebound was found in both groups at 1 and 3 months. Patients with thick phenotype were more propense to tissue rebound.
Phattarin et al., 2021 [9]	Controlled clinical trial	No	25	22–69 years old	Er,Cr:YSGG	G6 tip, 1.5 W, 7% W, 11% A	G6 tip, 3.5 W, 50% W, 40% A	Flapless laser-assisted crown lengthening AND conventional surgical technique	Plaque index (PI), gingival index (GI), relative gingival margin (RGM), relative bone level (RBL), biotype, attached gingival width, tooth mobility, and numeric rating scale (NRS) (days 1 and 7)	1 and 3 months	More stable GM in the laser group at 3-month follow-up
Min Yee et al., 2022 [17]	Case report	Not applicable	1	22 years old	Er:YAG	55 mJ/pulse, 20 Hz	70 mJ/Pulse, 20 Hz	Not applicable	Not specified	3 months and 1 year	Higher patient acceptance and similar outcomes to conventional surgery
Chang-Kai Chen et al., 2017 [18]	Case series	Not applicable	26	24–54 years old (mean age of 40.2 years)	Er:YAG	7 W, 200 mJ, 35 Hz, 87.5% W 0.6 mm conical quartz tip	Ostectomy: 1.5 W, 50 mJ, 30 Hz 75% W. To smoothen the bone edge: 150 mJ, 50 Hz, 7.5 W, 100% W	Not applicable (1 group with flapless surgery)	(A) GMLS (B) Planned restorative margin. (C) Crestal bone Distance from A to B and B to C PD, PI, GI, and BOP	3 and 6 months	No tissue rebound reported
Michael K et al., 2011 [19]	Case series	Not applicable	Not reported	Not reported	Er:YAG	3 W, 75 mJ, 40 Hz 600 µ tip chisel tip to remove troughs	1.5 W, 50 mJ, 30 Hz	Not applicable (opening the flap after flapless surgery)	GM position Health of attachment apparatus Patient satisfaction in terms of esthetics	6 months and 57% of the patients at 3 years.	Possible complications may occur with closed flap such as osseous troughs and root surface pitting.

**Table 2:** Grade of recommendation, level of evidence, and GRADE classification (grading the certainty of evidence) of the individual studies.

Study	Grade of Recommendation	Level of Evidence	GRADE Approach
Phattarin et al., 2021 [9]	B	2b	LOW (our confidence in the estimate of the effect is limited: the true effect may be substantially different from the estimate.)
Min Yee et al., 2022 [17]	C	4	Not applicable
Walid Altayeb et al., 2022 [7]	A	1b	MODERATE (we have moderate confidence in the effect estimate: the true effect is likely to be close to the effect estimate, but there is a possibility that it is substantially different.)
Chang-Kai Chen et al., 2017 [18]	C	4	Not applicable
Michael K et al., 2011 [19]	C	4	Not applicable
